# Supplementary figures and images for: Actin associates with actively elongating genes and binds directly to the Cdk9 subunit of P-TEFb
Source: J Biol Chem. 2024 Jan 30;300(3):105698. doi: 10.1016/j.jbc.2024.105698 (PMC10891344; doi:10.1016/j.jbc.2024.105698)

Figure S1

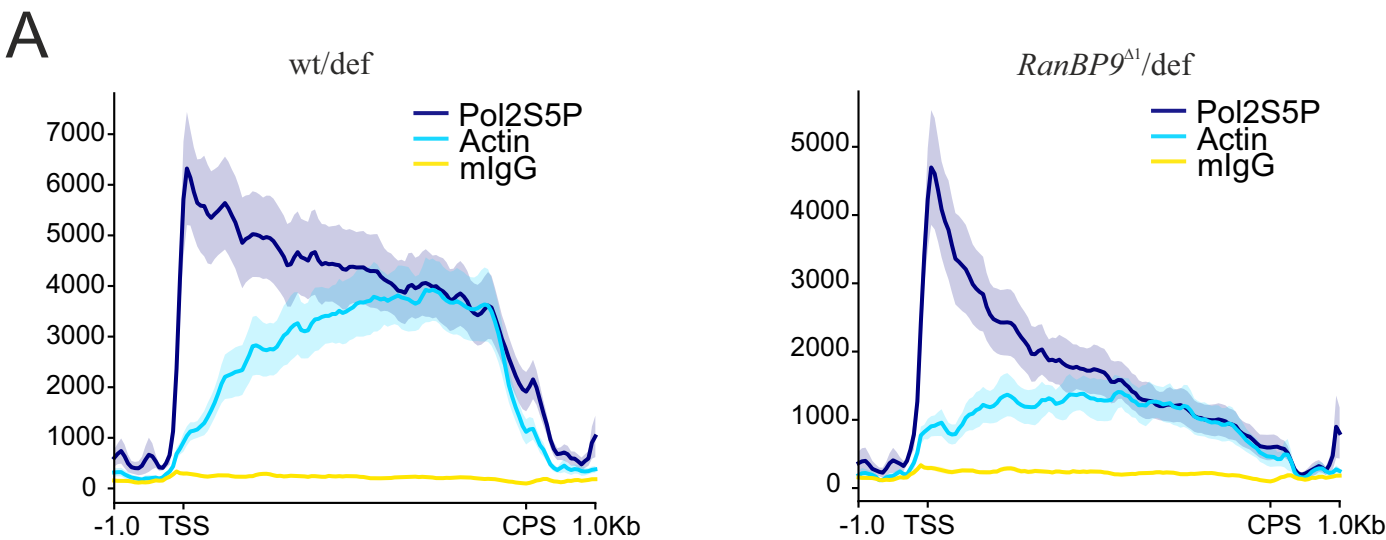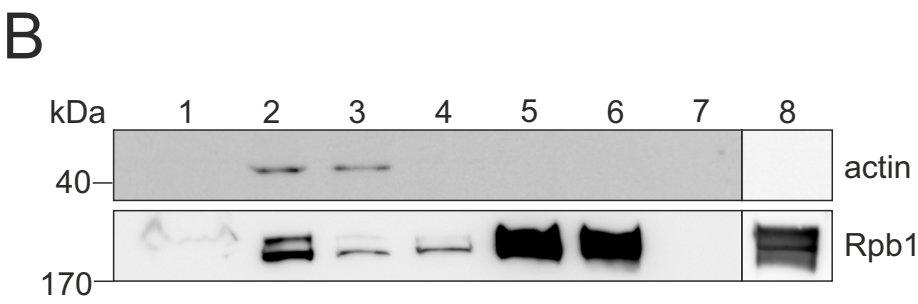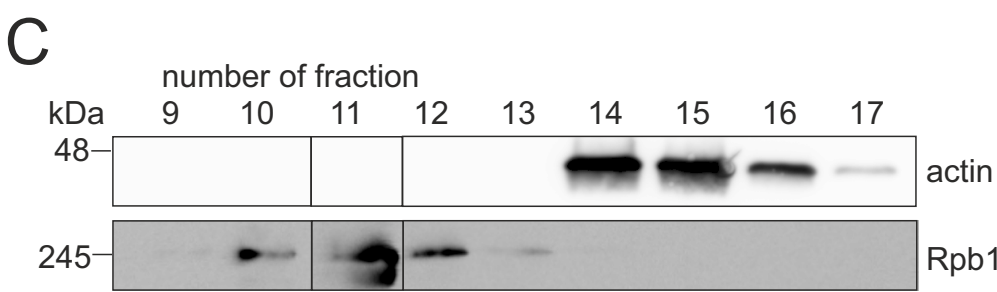

Supplement: Fig. S1 [file mmc1.pdf]
