# Supplementary material for: Actin associates with actively elongating genes and binds directly to the Cdk9 subunit of P-TEFb
Source: J Biol Chem. 2024 Jan 30;300(3):105698. doi: 10.1016/j.jbc.2024.105698 (PMC10891344; doi:10.1016/j.jbc.2024.105698)
Supplement: Supplementary data [file mmc2.pdf]

Original western blots - 1

Figure S1

B

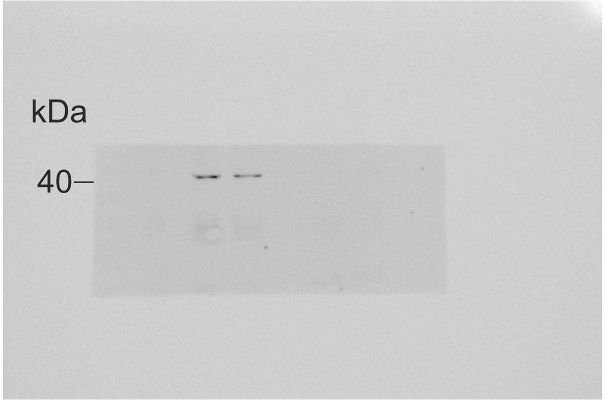

actin

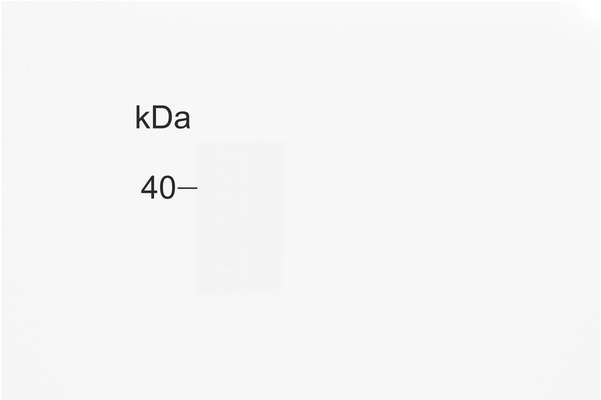

actin

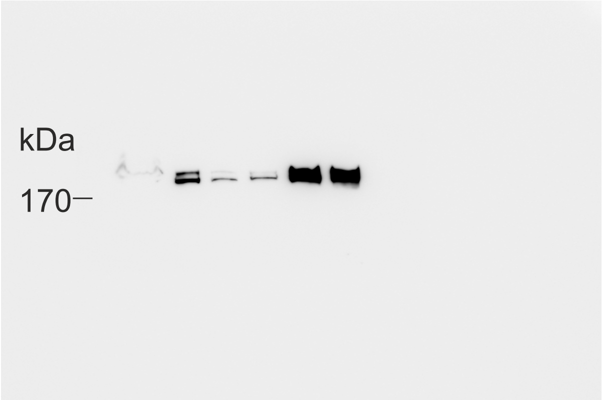

Rpb1

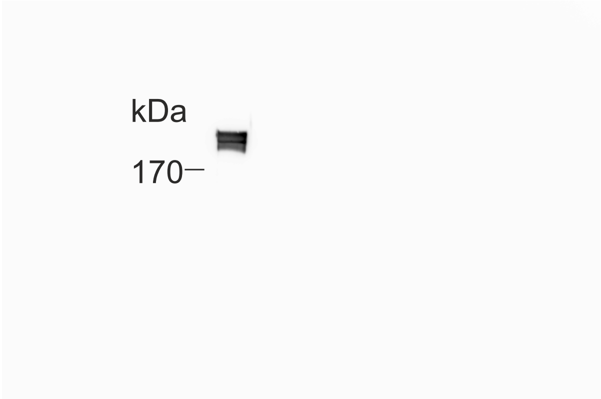

Rpb1

C

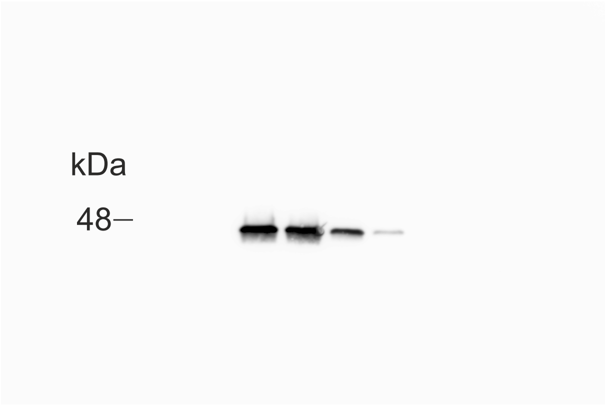

actin

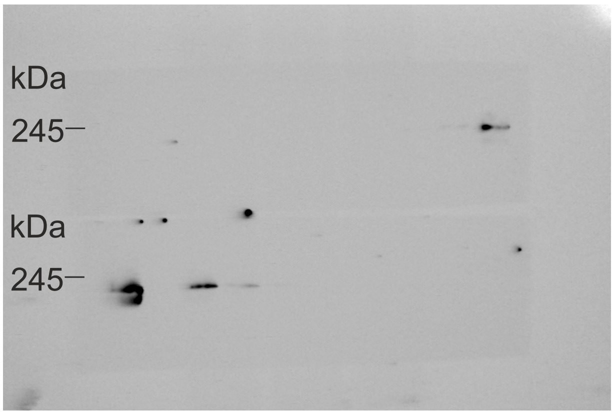

Rpb1

Original western blots - 2

Figure 2

A

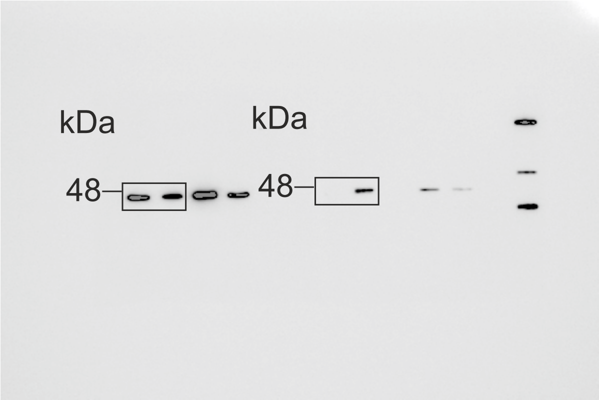

Flag-HRP

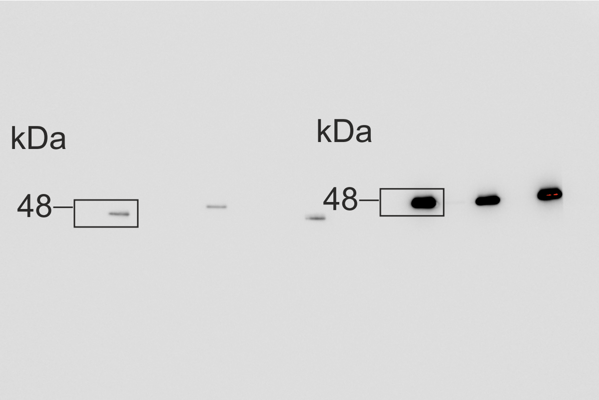

HA-HRP

B

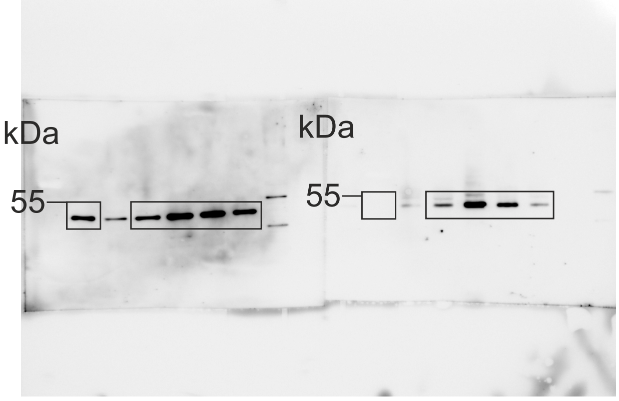

HA-HRP

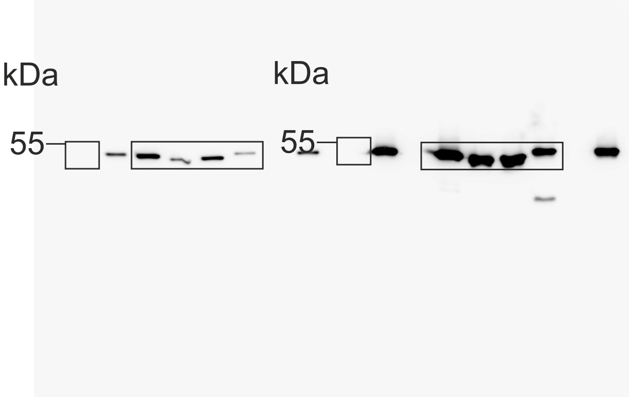

Flag-HRP

C

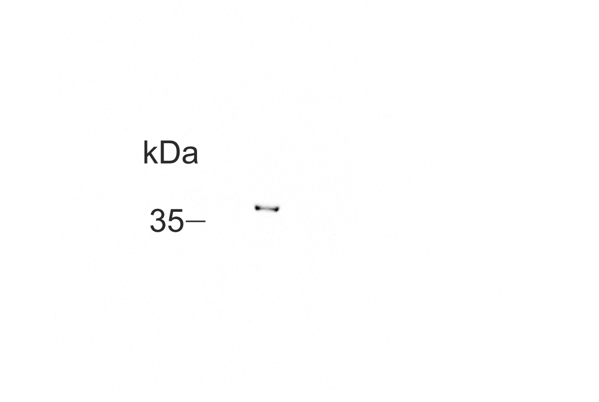

actin

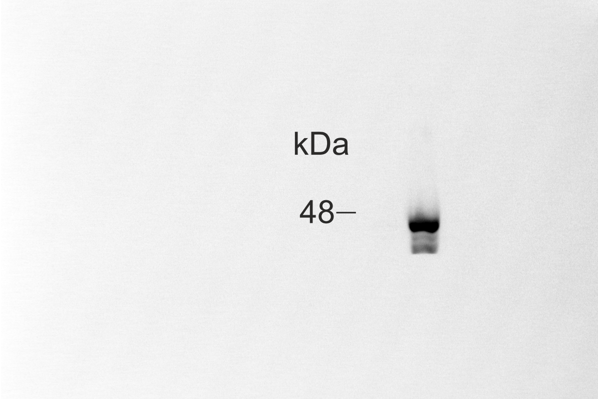

Cdk9

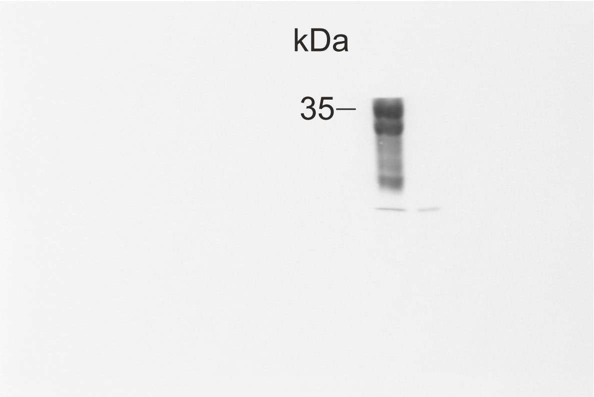

His-HRP

Original western blots - 3

Figure 5

B

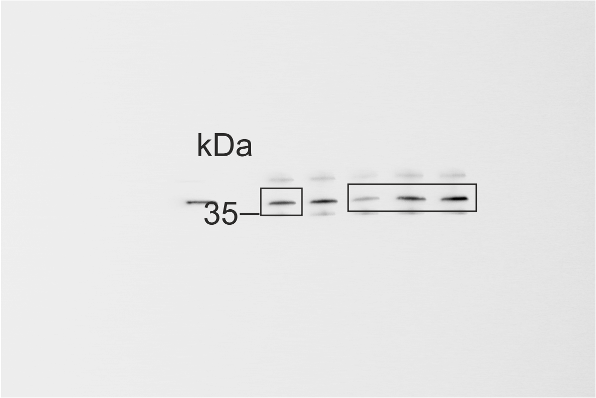

Cdk9

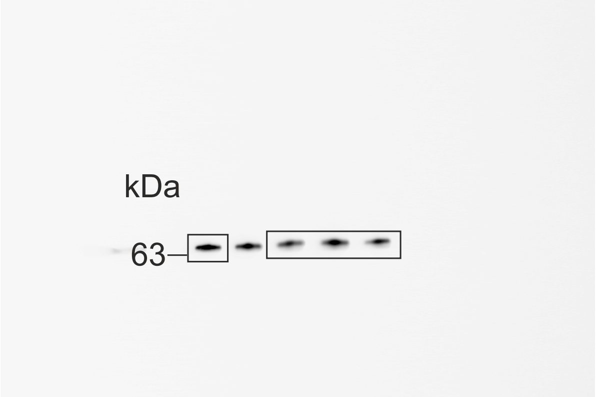

HEXIM1
